# Characterization and genome analysis of a butanol–isopropanol-producing *Clostridium beijerinckii* strain BGS1

**DOI:** 10.1186/s13068-018-1274-x

**Published:** 2018-10-11

**Authors:** Chen Zhang, Tinggang Li, Jianzhong He

**Affiliations:** Department of Civil and Environmental Engineering, National University of Singapore, Block E2-02-13, 1 Engineering Drive 3, Singapore, 117576 Singapore

**Keywords:** Isopropanol, Butanol, *Clostridium beijerinckii*, Genome, Secondary alcohol dehydrogenase

## Abstract

**Background:**

One of the main challenges of acetone–butanol–ethanol fermentation is to reduce acetone production with high butanol yield. Converting acetone into isopropanol is an alternative pathway to reduce fermentation by-products in the fermentation broth. Here, we aimed to cultivate a wild-type *Clostridium* strain with high isopropanol and butanol production and reveal its genome information.

**Results:**

*Clostridium beijerinckii* strain BGS1 was found to be capable of producing 10.21 g/L butanol and 3.41 g/L isopropanol, higher than previously known wild-type isopropanol–butanol-producing *Clostridium* species. Moreover, culture BGS1 exhibited a broad carbon spectrum utilizing diverse sugars such as arabinose, xylose, galactose, cellobiose, and sucrose, with 9.61 g/L butanol and 2.57 g/L isopropanol generated from 60 g/L sucrose and less amount from other sugars. Based on genome analysis, protein-based sequence of strain BGS1 was closer to *C. beijerinckii* NCIMB 8052, reaching 90.82% similarity, while compared to *C. beijerinckii* DSM 6423, the similarity was 89.53%. In addition, a unique secondary alcohol dehydrogenase (sAdhE) was revealed in the genome of strain BGS1, which distinguished it from other *Clostridium* species. Average nucleotide identity analysis identified strain BGS1 belonging to *C. beijerinckii*. The transcription profile and enzymatic activity of sAdhE proved its function of converting acetone into isopropanol.

**Conclusions:**

*Clostridium beijerinckii* strain BGS1 is a potential candidate for industrial isopropanol and butanol production. Its genome provides unique information for genetic engineering of isopropanol–butanol-producing microorganisms.

## Background

Isopropanol and butanol are widely used in a variety of industrial applications including solvent applications, chemical intermediates and biofuels. When serving as a biofuel, butanol can be used as a fuel additive or completely replace gasoline due to property similarities [1], while isopropanol can replace methanol for biodiesel synthesis with reduced biodiesel crystallization temperature [2]. Additionally, isopropanol can partially replace gasoline so as to increase octane number of the fuels [3]. However, given that both chemicals are mainly produced from non-renewable petroleum-based materials, microbial production of isopropanol and butanol from renewable feedstock becomes more attractive and cost effective [4].

As a traditional microbial way to produce butanol, acetone–butanol–ethanol (ABE) fermentation by *Clostridium* species can generate butanol, acetone and ethanol at a ratio of 6:3:1 typically [5]. However, the main by-product, acetone, is not preferred because of its high corrosiveness to engine and low energy density [6]. Although acetone production have been tried to be reduced by knock-out of acetoacetate decarboxylase (*adc*) gene, butanol reduction and acetate accumulation inevitably occurred with the acetone reduction [7]. In contrast, isopropanol–butanol–ethanol (IBE) fermentation by *Clostridium* species is a more preferred microbial pathway for both renewable butanol and isopropanol production without the undesirable acetone [8]. Compared to traditional ABE fermentation, IBE fermentation produces a component of isopropanol, an alcohol with slightly higher energy density than acetone (23.9 MJ/L vs 22.6 MJ/L), and the end-products of IBE mixture can be used directly as a green fuel in spark-ignition (SI) engines, usually with lower pollutant emissions and better engine performance than ABE mixture [9].

Among all the solvent-producing *Clostridium* strains, a few strains can produce butanol and isopropanol as major fermentation products, while most isopropanol-producing bacteria belong to *Clostridium beijerinckii* [10]. In general, most of the isopropanol-producing strains produce relatively low concentrations of butanol and isopropanol, less than 5.5 g/L and 1.5 g/L, respectively, due to the toxicity posed by butanol (> 7.4 g/L) [11]. To produce isopropanol with ABE-producing *Clostridium*, an attempt has been made to engineer secondary alcohol dehydrogenase (sAdhE) gene from *C. beijerinckii* NRRL B-593 into non-isopropanol producing *C. acetobutylicum* [6]. However, the successful conversion of acetone into isopropanol also caused reduction of butanol yield. To date, only one* sADH* gene from *C. beijerinckii* NRRL B-593 with limited genome information was identified, while this wild-type strain produced quite low titers (< 5 g/L) of butanol and isopropanol [12]. Very recently, *C. beijerinckii* DSM 6423 [13] was also found to possess *sAdhE* gene encoding enzymes for isopropanol and butanol production but with lower titers (< 10.7 g/L). Finding novel wild-type bacteria with higher isopropanol and butanol production as well as detailed genome information is of great interest for isopropanol and butanol biorefinery.

To broaden current knowledge of butanol and isopropanol production, this study aimed to (i) characterize an isopropanol–butanol-producing microorganism with high yield; (ii) analyze the genome of this strain and further identify functional genes responsible for isopropanol production.

## Results and discussion

### Isolation and characterization of *Clostridium* sp. strain BGS1

**Fig. 1:**
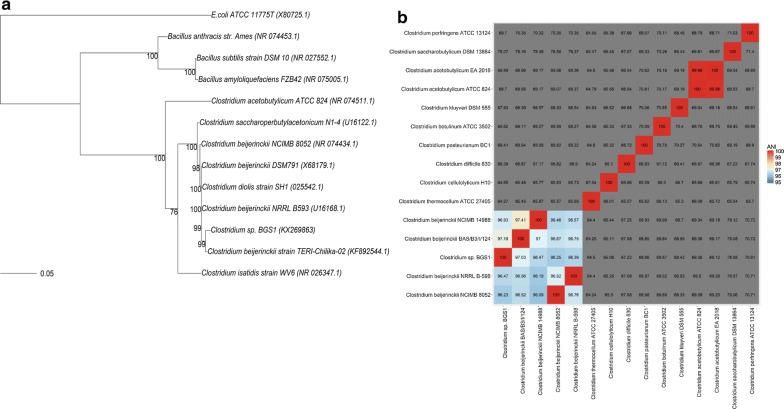
**a** Phylogenetic tree by maximum likelihood method based on the GTR + G model, **b** cluster heatmap of *Clostridium beijerinckii* BGS1 based on ANI calculation results

To discover novel isopropanol–butanol-producing microorganisms, soil samples from grass land were used as inocula for microcosm setup. After three transfers, four enriched cultures with butanol and isopropanol production were selected for further cultivation of individual colonies. Eight of well-grown colonies were picked up from deep agar medium spiked with 60 g/L glucose [14] and further transferred to fresh medium. Among all isolates, one culture designated BGS1 was identified to be capable of producing 3.88 g/L butanol and 1.07 g/L isopropanol with negligible amount of acetone and ethanol. When culture BGS1 was observed under light microscopy, the uniform rod-shaped morphology suggested the purity of the culture. The sequence of the 16S rRNA gene from culture BGS1 showed 99% identity to *C. beijerinckii* strain TERI-Chilika-02 based on Blast results. When comparing with 12 previously reported bacteria capable of producing butanol or isopropanol, culture BGS1 exhibited closer similarity to strains under *C. beijerinckii* (Fig. 1a). However, phylogenetic tree based on 16S rRNA gene can only identify microorganisms to genus level. Based on the genome information, average nucleotide identity (ANI) was applied to identify the species of strain BGS1 [15]. ANI of 95% between two bacterial genomes usually corresponds to 70% DNA–DNA hybridization value, from which it can confirm the two bacteria belonging to the same species [16]. As shown in Fig. 1b, cluster heatmap of ANI analysis clearly depicted that *Clostridium* sp. BGS1 clusters to *C. beijerinckii* species, of which all ANI values were higher than 96% of similarity but were lower than 80% of ANI values comparing with other *Clostridium* species. Hence, this isolate is designated *C. beijerinckii* strain BGS1.

*Clostridium beijerinckii* strain BGS1 distinguishes itself from other known *C. beijerinckii* strains by its high isopropanol production. As a typical *C. beijerinckii* strain, NCIMB 8052 mainly produced butanol and ethanol with acetone as a by-product, but was not able to produce isopropanol using glucose as a substrate [17]. Therefore, this newly isolated wild-type solvent-producing *C. beijerinckii* BGS1 broadens the pool of isopropanol–butanol-producing species [18].

### Optimization of fermentation conditions for enhanced butanol and isopropanol production

During the solvent fermentation, pH was a key factor influencing fermentation performance [5]. To optimize pH, five batch experiments were conducted at different pH conditions (5.0, 5.2, 5.5, 5.8, 6.5). pH was adjusted to a defined value by 3 M NaOH in every 12 h. After 3 days of incubation, culture BGS1 generated higher amount of butanol at pH 5.0 (5.00 g/L), pH 5.2 (4.46 g/L) and pH 5.5 (4.11 g/L), and lower amount at pH 5.8 (3.53 g/L) and pH 6.5 (1.93 g/L), while similar amounts of isopropanol were obtained at varied pH conditions. In addition, high pH (6.5) led to significantly increased acetic acid (6.36 g/L) and butyric acid (6.67 g/L) production in the fermentation broth, indicating that it was difficult for strain BGS1 to shift from acidogenesis to solventogenesis when pH is higher than 5.8. Therefore, maintaining a pH range from 5.0 to 5.5 is necessary to boost cell growth and early shift to solventogenesis. After pH optimization, 3 g/L yeast extract was supplemented to the medium, in which 6.50 g/L butanol and 3.01 g/L isopropanol were generated, the enhancement of which could be caused by yeast extract to maximize cell growth [19].

**Fig. 2:**
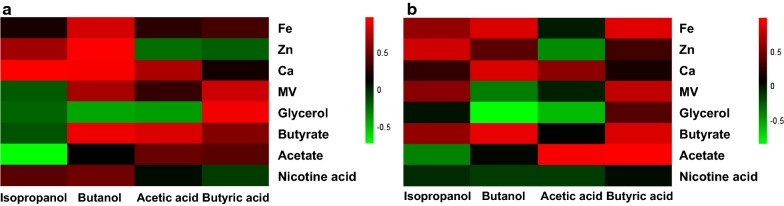
Heatmap of Pearson correlation coefficient of end-products and influence factors in medium containing **a** 60 g/L glucose, **b** 60 g/L sucrose

To further improve solvents production, eight factors potentially influencing fermentation performance were selected as supplement to the medium. The heatmap of Pearson correlation coefficient between end-products and factors is shown in Fig. 2a. Among all eight factors, supplementation of nicotine acid did not obviously increase butanol and isopropanol production compared to that of control. In comparison, Zn^2+^ and Ca^2+^ showed strong positive correlation with isopropanol; while Fe^2+^, Zn^2+^, Ca^2+^ and butyrate exhibited strong positive correlation with butanol. However, given that butyrate was negatively correlated with isopropanol production, it was not considered as a beneficial factor; thus, Fe^2+^, Zn^2+^ and Ca^2+^ were finally selected as enhancement factors to improve butanol and isopropanol production.

**Fig. 3:**
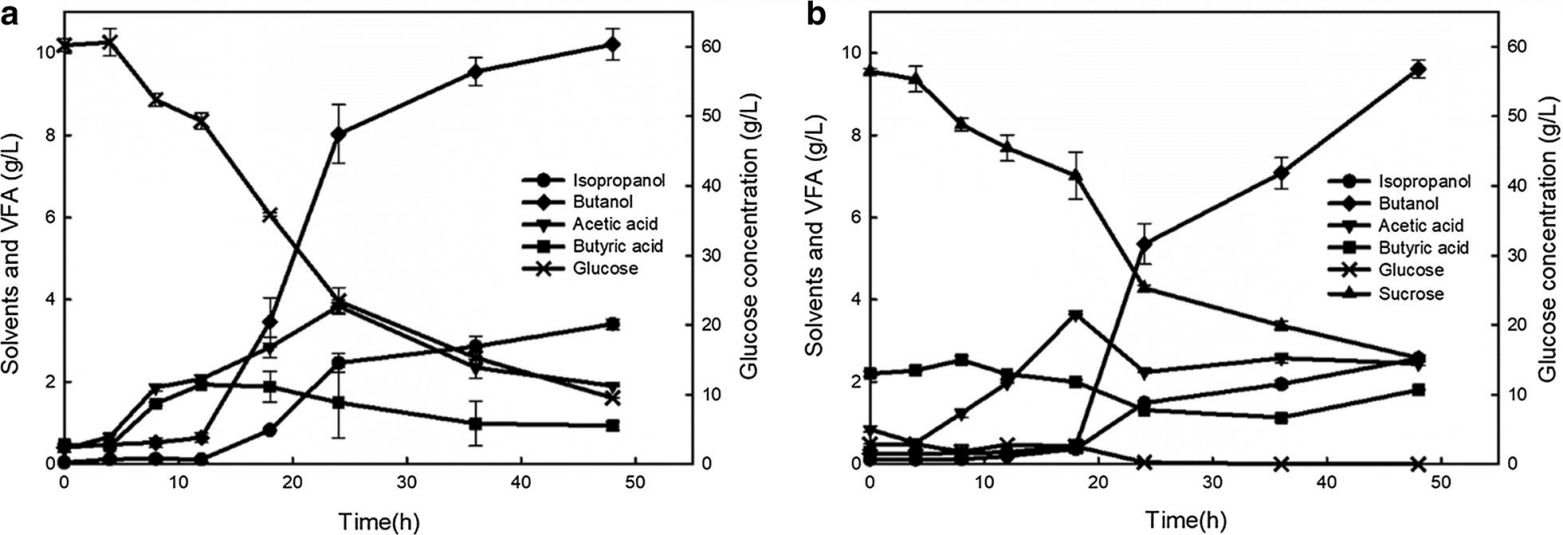
Optimized solvents production of *Clostridium beijerinckii* strain BGS1 in reduced mineral salts medium containing **a** 60 g/L glucose with 3 g/L yeast extract, 5 g/L CaCO_3_, 8 mg/L Zn^2+^ and 2 mg/L Fe^2+^, **b** 60 g/L sucrose with 3 g/L yeast extract, 1 g/L CaCO_3_, 3 g/L sodium butyrate, and 6 mg/L Fe^2+^

Subsequently, a response surface methodology (RSM) based on central composite design (CCD) by Design Expert version 8.0 was used to obtain optimized fermentation conditions [20]. According to the results of 20 experiments, the optimal condition was confirmed at Zn^2+^ of 8 mg/L, Fe^2+^ of 2 mg/L and CaCO_3_ of 5 g/L; thus, with 3 g/L yeast extract and the abovementioned three elements supplemented, culture BGS1 was capable of producing 10.21 g/L butanol and 3.41 g/L isopropanol (Fig. 3a). *Clostridium beijerinckii* BGS1 produced highest amount of butanol and third highest amount of isopropanol as compared to those of previously reported wild-type isopropanol–butanol-producing *Clostridium* (Table 1).

**Table 1:** Comparison of various wild-type isopropanol–butanol-producing wild-type strains

References	Substrate	Strain	Isopropanol (g/L)	Butanol (g/L)
P. G. Krouwel	glucose 57 g/L	*Clostridium butylicum* LMD 27.6	2.83	5.25
P. G. Krouwel	glucose 20 g/L	*Clostridium beijerinckii* LMD 27.6	1.33	3.95
J. S. CHEN	glucose 60 g/L	*Clostridium beijerinckii* VPI2968	0.12	3.06
J. S. CHEN	glucose 60 g/L	*Clostridium beijerinckii* VPI2968	0.59	3.32
J. S. CHEN	glucose 60 g/L	*Clostridium aurantibutylicum* NCIB 10659	0.60	4.24
J. S. CHEN	glucose 60 g/L	*Clostridium beijerinckii* B-593	0.48	4.57
Stephen F. Hiu	glucose 60 g/L	*Clostridium beijerinckii* NRRL B593	0.48	4.59
Stephen F. Hiu	glucose 60 g/L	*Clostridium beijerinckii* ATCC 6014	1.08	4.89
Stephen F. Hiu	glucose 60 g/L	*Clostridium beijerinckii* McClung 3081	1.56	6.00
Masatoshi Matsumura	cane molasses 50 g/L	*Clostridium isopropylicum* IAM 19239	4.60	8.30
Shrikant A. Survase	glucose 60 g/L	*Clostridium beijerinckii* DSM 6423	2.16	3.71
Truus de Vrije	glucose 40 g/L xylose 20 g/L	*Clostridium beijerinckii* NRRL B593	3.20	6.90
Sung Hun Youn	glucose 60 g/L	*Clostridium* sp. A1424	4.49	9.43
Ying Yang	glucose 30 g/L	*Clostridium beijerinckii* sp. optinoii	3.21	6.24
This study	glucose 60 g/L	*Clostridium beijerinckii* BGS1	3.41	10.21
This study	sucrose 60 g/L	*Clostridium beijerinckii* BGS1	2.51	9.79

### *Clostridium beijerinckii* strain BGS1 ferments diverse sugars

**Fig. 4:**
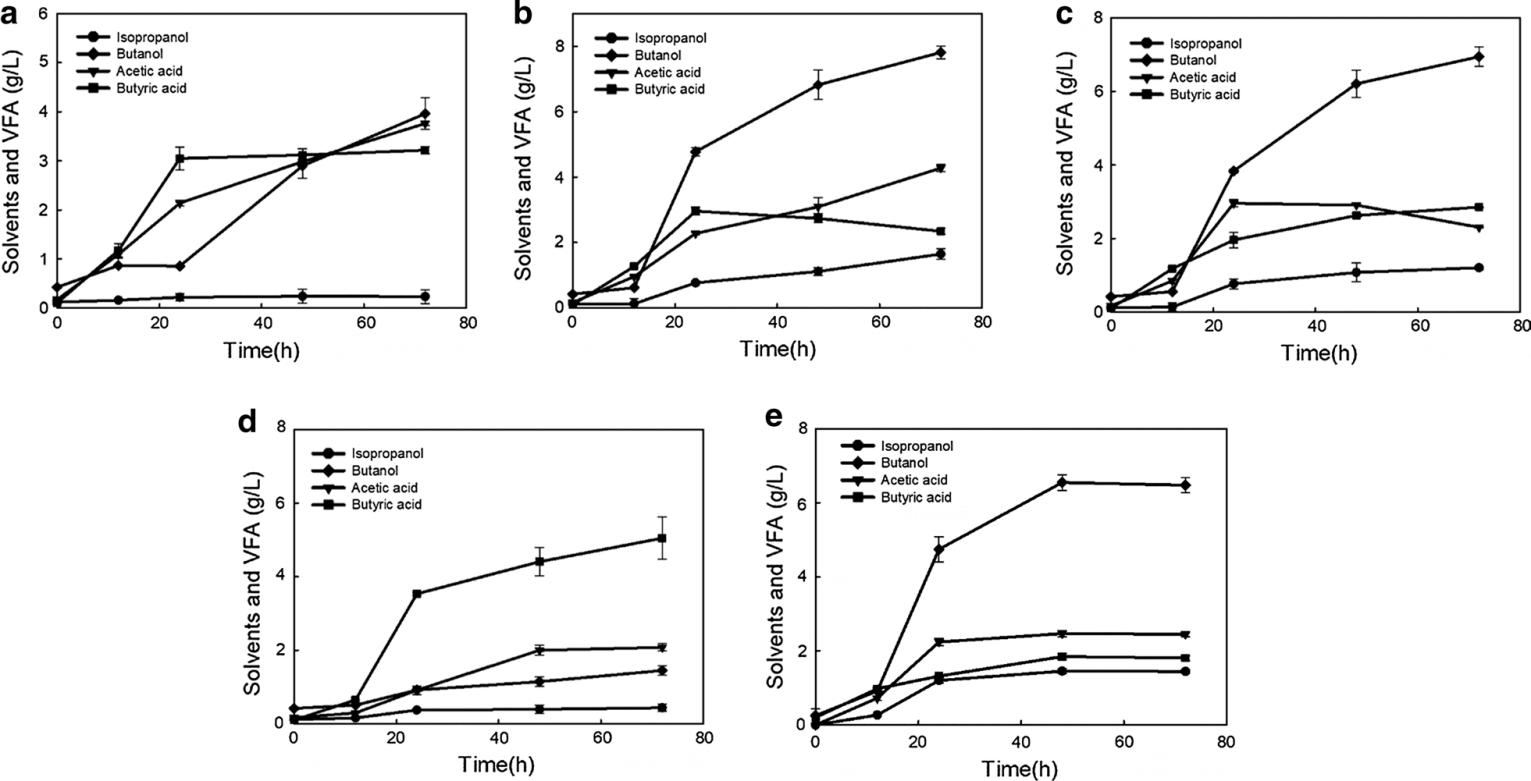
Solvents production by *Clostridium beijerinckii* strain BGS1 in reduced mineral salts medium containing 3 g/L yeast extract and **a** 60 g/L xylose, **b** 60 g/L arabinose, **c** 60 g/L cellobiose, **d** 60 g/L galactose, **e** 60 g/L sucrose

*Clostridium beijerinckii* strain BGS1 was assayed on its capability of fermenting various sugars (Fig. 4). For monosaccharides, in reduced mineral salts medium containing 3 g/L yeast extract, culture BGS1 was capable of producing 3.96, 7.81 or 1.45 g/L butanol, and 0.23, 1.65 or 0.44 g/L isopropanol when fed with 60 g/L xylose, arabinose or galactose, respectively. Although both xylose and arabinose belong to pentose, culture BGS1 can utilize arabinose much better than xylose, and produce similar amounts of butanol and isopropanol as compared to those with glucose as a carbon source. Strain BGS1 produced larger amount of acetic acid when fed with arabinose or xylose than glucose, indicating longer acidogenesis phase with pentose as a substrate. When galactose was used as a sole carbon source, culture BGS1 produced low concentrations of butanol and isopropanol, but with the highest butyric acid among six carbon sugars tested (Fig. 4d), suggesting an additional pathway from galactose to glycolysis involved in *C. beijerinckii* BGS1 [21]. Since galactose cannot be directly used for glycolysis, the low amount of butanol could be caused by Leloir pathway from galactose to glucose-6-phosphatase, which slowed down glycolysis step to the following acidogenesis and solventogenesis phases. Compared to another isopropanol- and butanol-producing strain *Clostridium* sp. A1424, strain BGS1 demonstrated its potential in utilizing pentose to produce more valued products since *Clostridium* sp. A1424 only produced acetic acid and butyric acid as final products from xylose and arabinose [22].

In addition to monosaccharides, strain BGS1 showed efficient utilization of oligosaccharides. Cellobiose, a disaccharide consisting of two glucose molecules, is derived from cellulose or cellulose-rich materials. When fed with cellobiose, culture BGS1 produced 6.94 g/L butanol and 1.21 g/L isopropanol (Fig. 4c). It is speculated that culture BGS1 possesses glucosidase genes responsible for hydrolyzing cellobiose to glucose. Additionally, culture BGS1 was capable of utilizing another disaccharide—sucrose to produce 6.48 g/L butanol and 1.44 g/L isopropanol (Fig. 4e). Since sucrose consists of glucose and fructose (furanoid-like xylose), strain BGS1 may prefer glucose to fructose due to the carbon catabolite repression [23], which led to slightly lower butanol generation from sucrose than from cellobiose. To enhance utilization of sucrose, optimization studies via spiking Fe^2+^, Zn^2+^, Ca^2+^, or butyrate to the fermentation medium with sucrose were conducted, of which results showed that Zn^2+^ did not affect butanol and isopropanol production. Collectively, culture BGS1 was capable of producing 9.61 g/L butanol and 2.57 g/L isopropanol from 60 g/L sucrose with addition of 1 g/L CaCO_3_, 3 g/L sodium butyrate, 6 mg/L Fe^2+^ to the medium (Fig. 3b). Since *C. beijerinckii* strain BGS1 can utilize multiple sugars, it is a potential candidate for industrial strain to ferment plant-based hydrolysate.

### Genome analysis of *C. beijerinckii* strain BGS1

To further explore the metabolic pathway of strain BGS1, the draft genome and functional annotation of strain BGS1 are illustrated here. The draft genome size of strain BGS1 is 5,880,896 bp with a low GC content of 29.71%. Based on annotation analysis, the genome of strain BGS1 contains 5223 predicted genes and the total length of genes is 4,721,271 bp, accounting for 80.28% of the whole draft genome. Additionally, the 5223 genes consist of 5008 coding sequences (CDSs), 99 tRNA genes, 28 rRNAs (including 11 5S rRNAs, 5 16S rRNAs, and 12 23S rRNAs) and 6 ncRNAs. Among the CDSs, subsystem functions according to RAST analysis indicate that a total of 704 CDSs are involved in the carbohydrates subsystems, in which fermentation subsystems and central carbohydrate metabolism contain 100 CDSs and 135 CDSs, respectively. For the related products and intermediates, 29 CDSs are related to fatty acid synthesis and 21 CDSs are related to NAD and NADP cofactor synthesis.

**Fig. 5:**
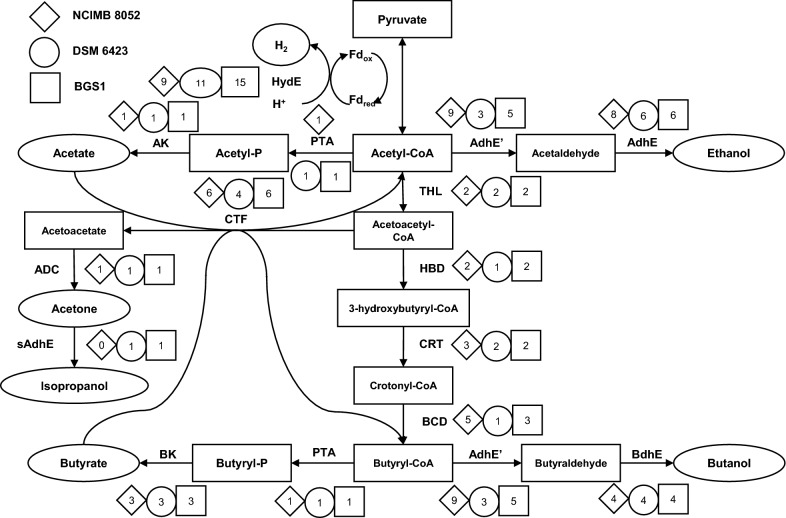
General solvents and VFAs production metabolic pathway from pyruvate in *Clostridium*. Abbreviation for the enzymes are: *scrA* sucrose-specific II component A, *scr6P* sucrose-6-phosphate hydrolase, *GPI* glucose-6-phosphate isomerase, *HydA* hydrogenase, *AdhE’* aldehyde dehydrogenase, *AdhE* alcohol dehydrogenase, *BdhE* butanol dehydrogenase, *PTA* phosphotransacetylase, *AK* acetate kinase, *CTF* CoA transferase, *HBD* 3-hydroxybutyryl-CoA dehydrogenase, *CRT* crotonase, *BCD* butyryl-CoA dehydrogenase, *BK* butyrate kinase, *ADC* acetoacetate decarboxylase, *THL* thiolase, *sAdhE* secondary alcohol dehydrogenase. The number means the corresponding gene number in whole genome

Further analysis of genome comparison revealed pathway specificity. A total of 54 CDSs are involved in the fermentation pathway [4] from pyruvate to final products including solvents (butanol and isopropanol) and VFAs (Fig. 5). Among 16 alcohol dehydrogenase-related CDSs, four *BdhE* genes encoding butanol dehydrogenase could be responsible for butanol production and one *sAdhE* gene encoding isopropanol dehydrogenase may result in the conversion from acetone into isopropanol. Compared to *C. beijerinckii* NCIMB 8052 and isopropanol–butanol-producing strain *C. beijerinckii* DSM 6423 [13], the number of CDSs related to metabolic pathway in BGS1 genome is more than that of DSM 6423 (a total of 45 CDSs) but is similar to that of NCIMB 8052 (a total of 56 CDSs). The most significant difference among these CDSs in the three strains is the *sAdhE* gene. The expression of *sAdhE* gene results in the production of isopropanol by BGS1 and DSM 6423 while in NCIMB 8052, the lack of *sAdhE* gene terminates further conversion of acetone into isopropanol. The *sAdhE* gene of BGS1 exhibited 90% (953/1056) similarity with that of DSM 6423. However, the function based on annotation showed that both are NADP-dependent and Zn-dependent alcohol dehydrogenases, which may correspond to isopropanol production improvement by Zn.

**Fig. 6:**
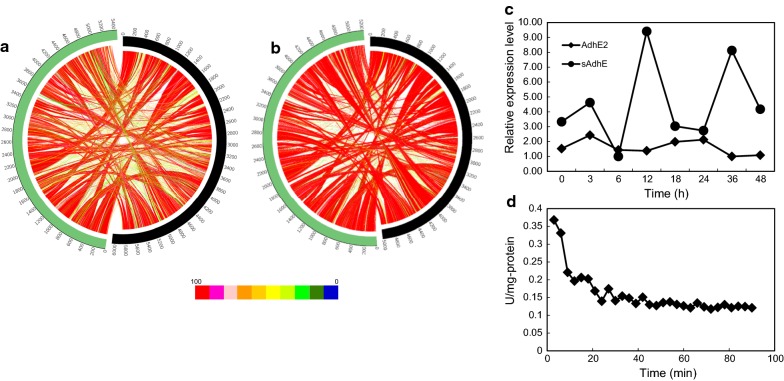
**a** Protein-based sequence comparison between BGS1 (green) and DSM 6423 (black). **b** Protein-based sequence comparison between BGS1 (green) and NCIMB 8052 (black). **c** Gene expression level of *sAdhE* and *AdhE2*. **d** Enzyme activity of sAdhE

In addition to nucleotide-based sequence comparison, protein-based comparison between *C. beijerinckii* BGS1 and two strains (strain DSM 6423 and NCIMB 8052) was also analyzed. As visualized in Fig. 6, similar protein sequence between BGS1 and reference strain was linked by curves and their similarity was distinguished by color range [24]. The larger yellow area in Fig. 6a demonstrated lower protein sequence similarity between BGS1 and DSM 6423 compared to the similarity between BGS1 and NCIMB 8052. Also, the average similarity between BGS1 and DSM 6423 was 89.53%, while between BGS1 and DSM 8052 was 90.82%. Additionally, protein function similarity among BGS, NCIMB 8052 and DSM 6423 provided different results (Table 2). BGS1 and DSM 6423 had 94.96% same protein function, while BGS1 and NCIMB 8052 only had 76.23% same protein function. The specific protein function was similar between BGS1 and DSM 6423. In contrast, NCIMB 8052 accounted for high percentage of specific protein function. Therefore, the two isopropanol–butanol-producing strains have closer protein function.

**Table 2:** Protein function similarity among *Clostridium beijerinckii* BGS1, DSM 6423 and NCIMB 8052

	BGS1 and DSM 6423	BGS1 and NCIMB 8052
Similarity	94.96%	76.23%
Unique protein in BGS1	2.55%	3.99%
Unique protein in DSM 6423	2.49%	–
Unique protein in NCIMB 8052	–	19.78%

To confirm whether the identified *sAdhE* gene was expressed in strain BGS1, the transcriptional expression of *sAdhE* gene were quantified by qPCR, Fig. 6c showed that the expression of *sAdhE* gene which reached to a peak after ~ 12 h of incubation. Interestingly, although expression of *sAdhE* gene gradually dropped after ~ 18-h incubation, it peaked again after ~ 36 h. Hence, it could be speculated that *sAdhE* gene was independently expressed at early-exponential and solventogenic phases. To prove this hypothesis, acetone was supplemented to culture. Results showed that acetone was converted into isopropanol and the amount of isopropanol increased twice. Given that gene expression cannot confirm the positive activity of enzyme, the enzymatic activity of sAdhE was conducted and it reached 0.12 U/mg protein (Fig. 6d). Additionally, one of the *AdhE* genes—*AdhE2*, was not obviously transcribed (Fig. 6c). Considering the fact that negligible ethanol was produced by culture BGS1, *AdhE2* gene may be responsible for ethanol production. Negligible expression of *AdhE2* resulted in non-ethanol production of culture BGS1.

## Conclusions

In this study, a wild-type *C. beijerinckii* BGS1 was identified to be capable of producing 10.21 or 9.61 g/L butanol and 3.41 or 2.57 g/L isopropanol from 60 g/L glucose or sucrose, respectively, higher than previously reported wild-type isopropanol–butanol-producing *Clostridium*. Moreover, the genome of strain BGS1 distinguished it from other *Clostridium* species. This study offers a promising butanol–isopropanol-producing strain due to its (i) capability of high butanol and isopropanol production from diverse saccharides; (ii) negligible ethanol and acetone production towards simple post-treatment process; (iii) novel *sAdhE* gene as a candidate for metabolic engineering of isopropanol production.

## Methods

### Culture isolation and characterization

Grassland soil samples from Mongolia were used as inocula for screening isopropanol–butanol-producing microorganisms. Reduced mineral salts medium was used as basic medium for isolation and batch fermentation, which contained 0.75 g/L of K_2_HPO_4_, 0.75 g/L of KH_2_PO_4_, 20 mM (2-*N*-morpholino ethanesulfonic acid) (MES), and 1 mL of trace element solution, 10 mL salt solution and 1 mL of Na_2_SeO_3_–Na_2_WO_4_ solution to 1 L solution with reductants of 0.2 mM Na_2_S, 0.2 mM l-cysteine and 0.5 mM dl-dithiothreitol [25]. After the medium was autoclaved for 20 min and cooled down to room temperature, a 50 mL medium with approximately 10 mL slurry soil and 60 g/L glucose were added to 160 mL bottles sealed with butyl stoppers. The microcosm was incubated at 35 °C and transferred five times for further isolation. The enriched culture was diluted and dispensed in 20 mL bottles containing the reduced mineral salts medium plus agar. The bottles were spiked with 60 g/L glucose and incubated at room temperature for individual colony growing. Individual colony was then picked and re-inoculated into 30 mL mineral salts medium fed with 60 g/L glucose. All colonies were randomly selected, and the products were monitored by GC-FID. Among several butanol-producing anaerobic bacteria, an isopropanol–butanol-producing strain (designated BGS1) was obtained.

Genomic DNA of culture BGS1 was extracted and purified with DNeasy Tissue Kit (Qiagen, Germany) according to the manufacture’s protocol. The 16S rRNA gene of strain BSG1 was amplified with a pair of universal bacterial primer 8F (5′-AGAGTTTGATCCTGGCTCAG-3′) and 1392R (5′-ACGGGCGGTGTGT-3′) [25]. The obtained PCR products were purified with a PCR Purification Kit (Qiagen, Germany) and sequenced using an ABI DNA sequencer (Applied Biosystems, USA). The obtained 16S rRNA gene sequence was analyzed by basic local alignment search tool (BLAST). The nucleotide sequence of culture BGS1 was deposited in GenBank under an accession number KX269863.

### Growth condition and optimization of culture BGS1

Time-course studies of culture BGS1 were conducted in the mineral salts medium as described above. Cultures for inoculation were grown in the medium containing 60 g/L glucose or other carbon sources (monosaccharides, disaccharides and polysaccharides) for ~ 24 h. Then, 6 mL inocula was added into 54 mL mineral salts medium in 160 mL serum bottles, in which the culture was incubated in a shaker at 35 °C with a shaking speed of 130 rpm. NaOH (3 M) was applied to adjust pH to 5.5. To optimize the growth condition for culture BGS1, different pH (4.5, 5.0, 5.2, 5.5, 5.8, 6.5) and nine ingredients possibly influencing fermentation performance (nicotinic acid, acetic acid, butyric acid, yeast extract, Ca^2+^, Fe^2+^, Zn^2+^, methyl viologen, glycerol) were tested. Nicotine acid (0–20 mg/L) and butyrate (0–5 g/L) are the precursors of reducing cofactor NADH and butanol, respectively. Supplementation of acetate (0–5 g/L) was able to induce early solventogenesis phase to produce solvents [26]. CaCO_3_ (0–7 g/L) had ability to enhance butanol tolerance by stabilizing membrane proteins and increased buffering capacity [27]. Glycerol (0–120 g/L) supplemented to medium containing glucose was reported to enhance cell growth and butanol production [28]. Zn^2+^ (0–10 mg/L), Fe^2+^ (0–8 mg/L) and methyl viologen (MV) (0–10 mg/L) were considered as co-factors to improve solvents production [29]. Experiments were carried out in duplicates.

### Data analysis methods

To acquire optimal fermentation conditions influenced by various factors, analysis was completed by response surface method (RSM) [20]. Concentrations of butanol and isopropanol were selected as dependent variables and influencing factors were independent variables. Central composite design (CCD) was chosen to apply RSM using Design Expert version 8.0 [30]. A second-order polynomial equation for the response variables was built:

1 $$Y_{\text{i}} = \beta_{0} + \sum \beta_{\text{i}} x_{\text{i}} + \sum \beta_{\text{ii}} x_{\text{ii}}^{2} + \int {\beta_{\text{ij}} x_{\text{i}} x_{\text{j}} } ,$$

where *Y*_i_ is the predicted response; *x*_i_, *x*_j_ are independent variables which influence the dependent variable *Y*; *β*_0_ is the offset term; *β*_i_ is the ith linear coefficient; *β*_ii_ is the ith quadratic coefficient; and *β*_ij_ is the ijth interaction coefficient.

All statistical analysis, phylogenetic tree, heatmap analysis and species cluster were addressed with R and the Bioconductor [31, 32]. Phylogenetic tree was analyzed using phangorn package [33]. Best tree model was selected by “bestmodel” command and phylogenetic tree was built by 1000 bootstrap. Pearson correlation coefficient was calculated on between duplicate factors concentration and concentrations of two products (butanol and isopropanol). Heatmap of Pearson correlation coefficient was drawn by pheatmap package [34]. Hierarchical cluster analysis was determined by the distance of all species from results of ANI analysis, which formed the ANI cluster heatmap.

### Analytic methods

Fermentation broth (1 mL) was centrifuged at 12,000×*g* for 10 min at 4 °C and the resultant supernatant was stored at − 20 °C until further analysis of the fermentation products. The solvents and fatty acids were measured by a gas chromatography (GC, model 7890A; Agilent Technologies, U.S.A.) equipped with a Durabond (DB)-WAXetr column (30 m × 0.25 mm × 0.25 µm; J&W, U.S.A.) and a flame ionization detector (FID). The oven temperature was initially held at 60 °C for 2 min, increased at 15 °C/min to 230 °C, and held for 1.7 min. Helium was used as the carrier gas with a column flow rate of 1.5 mL/min. Five-point standard curves were determined by standard solutions containing isopropanol, butanol, ethanol, acetone, butyric acid and acetic acid with concentrations ranging from 2 to 16 g/L. A high-performance liquid chromatograph (HPLC, model 1260 Infinity; Agilent Technologies, U.S.A) was used to measure concentrations of glucose and xylose with an Agilent Zorbax Carbohydrate Analysis column (4.6 mm × 150 mm, 0.5 μm) and a refractive index detector (RID). Supernatant samples (10 μL) as described above were injected into the column with a mobile phase (75% acetonitrile and 25% water) at a flow rate of 1 mL/min and oven temperature of 40 °C. A serial diluted glucose and sucrose ranging from 1 to 100 g/L were prepared for standard curves.

### Enzyme activity test

All tests were conducted under anaerobic conditions. Crude cell extracts were prepared from 10 mL of the culture. After centrifugation at 14,000 rpm at 4 °C for 10 min, the cell pellets were resuspended in 0.5 mL of ice-cold TE buffer (10 mM Tris–HCl, 5 mM EDTA, pH 7.5). Lysis was achieved by ultrasonication on ice for 15 min using a 20 kHz ultrasonicator (VCX 130, Sonics & Materials Inc., CT, USA) with the following repeated steps: 5 s of sonication with a 10 s interval, set at 50% amplitude. The collected lysate was then centrifuged at 14,000 rpm at 4 °C for 20 min for cell debris removal. The supernatant was used for enzymatic activity test and stored in 1 mL solution tube. DC protein assay Kit (BioRad, USA) was used to determine protein concentration of cell extracts. The standard curve of NADPH was prepared using diluted 1 mM NADPH Standard 1:5 to 0.2 mM NADPH by adding 20 µL NADPH Standard to 80 µL Assay Buffer. Then, 10, 20, 30, 40 µL of the 0.2 mM NADPH Standard were added into a series of wells in a 96-well plate to generate 2, 4, 6, 8 nmole/well NADPH standards. Finally, the volume was adjusted to 50 µL with ADH1-NADP Assay Buffer. sAdhE activity was assayed by monitoring the oxidation of NADPH at 340 nm. The NADPH-dependent reaction was examined at pH 8.0 under the following conditions: 0.4 mM NADPH, 50 mM acetone, 35 mM Tris chloride (pH 8.0), and crude extract (60–600 μg of protein). Final reaction volume was 600 μL. The change in absorbance of blank reactions without acetone was subtracted from the total. The reaction was initiated by the addition of the cell extract. One unit of specific enzymatic activity was defined as the amount of enzyme which oxidized 1 umol NADPH per minute per milligram of protein at 30 °C under the given conditions. All values of enzymatic activity test were averaged values of at least two independent extract procedures.

### Genome sequence analysis

The genomic DNA of *C. beijerinckii* BGS1 was sequenced using high-throughput Illumina HiSeq2000 sequencing platform in Beijing Genomics Institute (BGI), Shenzhen, China. The platform generated 17,509,102 reads with 488 insert sizes, totaling 8544 Mbp and providing 90-fold coverage. To determine the difference between sequenced species and reference species through average depth and coverage ratio calculation, the raw reads were firstly aligned to reference sequence (*C. beijerinckii* NCIMB 8052) by SOAPaligner (version 2.21). Then, the filtered reads were assembled using the SOAPdenovo program (version 2.04), producing a genome size of 5,885,271 bp with 117 contigs and an *N*_*50*_ length of 192,477 bp. Finally, 105 scaffolds with a maximum length of 414,297 bp were obtained through combining these contigs after contamination screening by NCBI. The draft genome was initially annotated by NCBI Prokaryotic Genome Annotation Pipeline (See http://www.ncbi.nlm.nih.gov/genome/annotation_prok/). In addition, Rapid Annotation using Subsystems Technology (RAST) server was used to depict the subsystem distribution of functional annotation results and also analyze the difference of protein sequences [35]. In RAST server, the tRNA genes were identified by tRNAscan-SE [36] and the rRNA encoding genes were identified using a tool “search_for_RNAs” [37]. Putative protein-encoding genes were initially called by GLIMMER3 [38] and then analyzed by BLASTP and BLASTX [39]. Lastly, the predicated functions of protein-encoding genes were annotated through a set of subsystem-based *FIGfams* protein database [37].

Average nucleotide identity (ANI), a measure of the pairwise average nucleotide identity shared between two genomes, was multi-analyzed using Jspecies software [40]. 15 genome sequences of *Clostridium* species including BGS1 were selected for ANI calculation based on MUMmer package [41]. All genome sequences are fragmented into 1020 bp regions which are then compared with those of the other species.

To analyze the expression of two specific genes encoding secondary alcohol dehydrogenase (sAdhE) and alcohol dehydrogenase (AdhE2) responsible for isopropanol and ethanol production, respectively, quantitative real-time PCR (qPCR) was used in this study. Primers used in this experiment are shown in Table 3. At every sampling time, 1 mL of cultures was harvested by centrifuging at 14,000 rpm and 4 °C for 10 min. Total RNA was then extracted from the resulting pellets using a combined Trizol and RNeasy Mini Kit (Qiagen, Hilden, Germany) according to manufacturer’s protocol. RNase-free DNase Kit (Qiagen, Hilden, Germany) was used to remove potentially environmental genomic DNA. Corresponding cDNA was generated from extracted RNA using a High Capacity cDNA Reverse Transcription Kit (Applied Biosystems) with RNase inhibitor and random hexanucleotide primer (both from Promega). cDNA samples were used as DNA templates for qPCR amplification using an ABI 7500 Fast real-time PCR system (ABI, Foster City, CA) with QuantiTect SYBR Green Kit (Qiagen, GmBH, Germany). To calculate the relative abundance, cycle to threshold value (Ct value) of each target gene was normalized to the abundance of the 16S rRNA gene for comparison. The final results were expressed as fold copies by normalizing lowest point of the relative abundance.

**Table 3:** Primers for genes *sAdhE* and *AdhE2*

Gene	Primer forward	Primer reverse
*sAdhE*	5′-AAT CAT GGC AGG TGG AGG TG-3′	5′-TTT CTG CTC GTA AAC GCC CT-3′
*AdhE2*	5′-TAT TAG CCA CTG GAG GTC CAG G-3′	5′-CGC AAA CTA CCC CGT TAT CAA-3′

#### Nucleotide sequence accession number

This Whole Genome Shotgun project has been deposited at DDBJ/ENA/GenBank under accession number MBAF00000000.

## Abbreviations

ABE: acetone–butanol–ethanol
adc: acetoacetate decarboxylase
AdhE: alcohol dehydrogenase
ANI: average nucleotide identity
BdhE: butanol dehydrogenase
CDSs: coding sequences
IBE: isopropanol–butanol–ethanol
MV: methyl viologen
NAD: nicotinamide adenine dinucleotide
NADP: nicotinamide adenine dinucleotide phosphate
RAST: Rapid Annotation using Subsystems Technology
RSM: response surface method
sAdhE: secondary alcohol dehydrogenase
VFAs: volatile fatty acids

## References

[1] Gu Y, Jiang Y, Wu H, Liu X, Li Z, Li J, Xiao H, Shen Z, Dong H, Yang Y (2011). Economical challenges to microbial producers of butanol: feedstock, butanol ratio and titer. Biotechnol J.

[2] Lee I, Johnson LA, Hammond EG (1995). Use of branched-chain esters to reduce the crystallization temperature of biodiesel. J Am Oil Chem Soc.

[3] Rassadin V, Shlygin OY, Likhterova N, Slavin V, Zharov A (2006). Problems in production of high-octane, unleaded automotive gasolines. Chem Technol Fuels Oils.

[4] Cho C, Jang Y-S, Moon HG, Lee J, Lee SY (2015). Metabolic engineering of clostridia for the production of chemicals. Biofuels Bioprod Biorefin.

[5] Jones DT, Woods DR (1986). Acetone–butanol fermentation revisited. Microbiol Rev.

[6] Lee J, Jang Y-S, Choi SJ, Im JA, Song H, Cho JH, Papoutsakis ET, Bennett GN, Lee SY (2012). Metabolic engineering of *Clostridium acetobutylicum* ATCC 824 for isopropanol–butanol–ethanol fermentation. Appl Environ Microbiol.

[7] Lehmann D, Hönicke D, Ehrenreich A, Schmidt M, Weuster-Botz D, Bahl H, Lütke-Eversloh T (2012). Modifying the product pattern of *Clostridium acetobutylicum*. Appl Microbiol Biotechnol.

[8] Osburn O, Brown R, Werkman C (1937). The butyl alcohol–isopropyl alcohol fermentation. J Biol Chem.

[9] Li Y, Meng L, Nithyanandan K, Lee TH, Lin Y, Chia-fon FL, Liao S (2016). Combustion, performance and emissions characteristics of a spark-ignition engine fueled with isopropanol–*n*-butanol–ethanol and gasoline blends. Fuel.

[10] Chen J-S, Hiu SF (1986). Acetone–butanol–isopropanol production by *Clostridium beijerinckii* (synonym, *Clostridium butylicum*). Biotech Lett.

[11] Lee SY, Park JH, Jang SH, Nielsen LK, Kim J, Jung KS (2008). Fermentative butanol production by *Clostridia*. Biotechnol Bioeng.

[12] George HA, Johnson JL, Moore WE, Holdeman LV, Chen JS (1983). Acetone, isopropanol, and butanol production by *Clostridium beijerinckii* (syn. *Clostridium butylicum*) and *Clostridium aurantibutyricum*. Appl Environ Microbiol.

[13] Máté de Gérando H, Wasels F, Bisson A, Clement B, Bidard F, Jourdier E, López-Contreras AM, Lopes Ferreira N (2018). Genome and transcriptome of the natural isopropanol producer *Clostridium beijerinckii* DSM6423. BMC Genomics.

[14] Miller NJ, Garrett OW, Prickett PS (1939). Anaerobic technique—a modified deep agar shake. J Food Sci.

[15] Goris J, Konstantinidis KT, Klappenbach JA, Coenye T, Vandamme P, Tiedje JM (2007). DNA–DNA hybridization values and their relationship to whole-genome sequence similarities. Int J Syst Evol Microbiol.

[16] Stackebrandt E, Goebel B (1994). Taxonomic note: a place for DNA-DNA reassociation and 16S rRNA sequence analysis in the present species definition in bacteriology. Int J Syst Evol Microbiol.

[17] Lee S-M, Cho MO, Park CH, Chung Y-C, Kim JH, Sang B-I, Um Y (2008). Continuous butanol production using suspended and immobilized *Clostridium beijerinckii* NCIMB 8052 with supplementary butyrate. Energy Fuels.

[18] Chen J-S (1995). Alcohol dehydrogenase: multiplicity and relatedness in the solvent-producing *clostridia*. FEMS Microbiol Rev.

[19] Li M, Liao X, Zhang D, Du G, Chen J (2011). Yeast extract promotes cell growth and induces production of polyvinyl alcohol-degrading enzymes. Enzyme Res.

[20] Bezerra MA, Santelli RE, Oliveira EP, Villar LS, Escaleira LA (2008). Response surface methodology (RSM) as a tool for optimization in analytical chemistry. Talanta.

[21] Sellick CA, Campbell RN, Reece RJ, Jeon KW (2008). Chapter 3 Galactose metabolism in yeast—structure and regulation of the leloir pathway enzymes and the genes encoding them. International review of cell and molecular biology.

[22] Youn SH, Lee KM, Kim K-Y, Lee S-M, Woo HM, Um Y (2016). Effective isopropanol–butanol (IB) fermentation with high butanol content using a newly isolated *Clostridium* sp. A1424. Biotechnol Biofuels.

[23] Görke B, Stülke J (2008). Carbon catabolite repression in bacteria: many ways to make the most out of nutrients. Nat Rev Microbiol.

[24] Krzywinski M, Schein J, Birol I, Connors J, Gascoyne R, Horsman D, Jones SJ, Marra MA (2009). Circos: an information aesthetic for comparative genomics. Genome Res.

[25] Xin F, He J (2013). Characterization of a thermostable xylanase from a newly isolated *Kluyvera* species and its application for biobutanol production. Bioresour Technol.

[26] Li R-D, Li Y-Y, Lu L-Y, Ren C, Li Y-X, Liu L (2011). An improved kinetic model for the acetone–butanol–ethanol pathway of *Clostridium acetobutylicum* and model-based perturbation analysis. BMC Syst Biol.

[27] El Kanouni A, Zerdani I, Zaafa S, Znassni M, Loutfi M, Boudouma M (1998). The improvement of glucose/xylose fermentation by Clostridium acetobutylicum using calcium carbonate. World J Microbiol Biotechnol.

[28] Ujor V, Agu C, Gopalan V, Ezeji T (2014). Glycerol supplementation enhances furfural detoxification by *Clostridium beijerinckii* during butanol fermentation. Appl Microbiol Biotechnol.

[29] Ujor V, Okonkwo C, Ezeji TC (2016). Unorthodox methods for enhancing solvent production in solventogenic *Clostridium* species. Appl Microbiol Biotechnol.

[30] Merrill MD (1987). An expert system for instructional design. IEEE Expert.

[31] R Core Team (2017). R: a language and environment for statistical computing.

[32] Huber W, Carey VJ, Gentleman R, Anders S, Carlson M, Carvalho BS, Bravo HC, Davis S, Gatto L, Girke T (2015). Orchestrating high-throughput genomic analysis with Bioconductor. Nat Methods.

[33] Schliep KP (2011). Phangorn: phylogenetic analysis in R. Bioinformatics.

[34] Kolde R: pheatmap: Pretty Heatmaps. R package version 1.0.8.; 2015. https://CRAN.R-project.org/package=pheatmap

[35] Overbeek R, Olson R, Pusch GD, Olsen GJ, Davis JJ, Disz T, Edwards RA, Gerdes S, Parrello B, Shukla M (2014). The SEED and the rapid annotation of microbial genomes using subsystems technology (RAST). Nucleic Acids Res.

[36] Lowe TM, Eddy SR (1997). tRNAscan-SE: a program for improved detection of transfer RNA genes in genomic sequence. Nucleic Acids Res.

[37] Aziz RK, Bartels D, Best AA, DeJongh M, Disz T, Edwards RA, Formsma K, Gerdes S, Glass EM, Kubal M (2008). The RAST Server: rapid annotations using subsystems technology. BMC Genomics.

[38] Delcher AL, Harmon D, Kasif S, White O, Salzberg SL (1999). Improved microbial gene identification with GLIMMER. Nucleic Acids Res.

[39] Altschul SF, Madden TL, Schäffer AA, Zhang J, Zhang Z, Miller W, Lipman DJ (1997). Gapped BLAST and PSI-BLAST: a new generation of protein database search programs. Nucleic Acids Res.

[40] Richter M, Rosselló-Móra R (2009). Shifting the genomic gold standard for the prokaryotic species definition. Proc Natl Acad Sci.

[41] Kurtz S, Phillippy A, Delcher AL, Smoot M, Shumway M, Antonescu C, Salzberg SL (2004). Versatile and open software for comparing large genomes. Genome Biol.

